# Manual segmentation of the paraventricular nucleus of the hypothalamus and the dorsal and ventral bed nucleus of stria terminalis using multimodal 7 Tesla structural MRI: probabilistic atlases for a stress-control triad

**DOI:** 10.1007/s00429-023-02713-z

**Published:** 2023-10-09

**Authors:** Brandon M. Sibbach, Helmet T. Karim, Daniel Lo, Nithya Kasibhatla, Tales Santini, Jessica C. Weber, Tamer S. Ibrahim, Layla Banihashemi

**Affiliations:** 1 Department of Psychiatry, University of Pittsburgh, Pittsburgh, PA 15213 USA; 2 Department of Bioengineering, University of Pittsburgh, Pittsburgh, PA 15213 USA

**Keywords:** Paraventricular nucleus of hypothalamus, Bed nucleus of stria terminalis, Manual segmentation, Probabilistic atlas, Stress, Anxiety

## Abstract

The paraventricular nucleus of the hypothalamus (PVN) is uniquely capable of proximal control over autonomic and neuroendocrine stress responses, and the bed nucleus of the stria terminalis (BNST) directly modulates PVN function, as well as playing an important role in stress control itself. The dorsal BNST (dBNST) is predominantly preautonomic, while the ventral BNST (vBNST) is predominantly viscerosensory, receiving dense noradrenergic signaling. Distinguishing the dBNST and vBNST, along with the PVN, may facilitate our understanding of dynamic interactions among these regions. T1-weighted MPRAGE and high resolution gradient echo (GRE) modalities were acquired at 7T. GRE was coregistered to MPRAGE and segmentations were performed in MRIcroGL based on their Atlas of the Human Brain depictions. The dBNST, vBNST and PVN were manually segmented in 25 participants; 10 images were rated by 2 raters. These segmentations were normalized and probabilistic atlases for each region were generated in MNI space, now available as resources for future research. We found moderate–high inter-rater reliability [*n* = 10; Mean Dice (SD); PVN = 0.69 (0.04); dBNST = 0.77 (0.04); vBNST = 0.62 (0.04)]. Probabilistic atlases were reverse normalized into native space for six additional participants that were segmented but not included in the original 25. We also found moderate to moderate–high reliability between the probabilistic atlases and manual segmentations [*n* = 6; Mean Dice (SD); PVN = 0.55 (0.12); dBNST = 0.60 (0.10); vBNST = 0.47 (0.12 SD)]. By isolating these hypothalamic and BNST subregions using ultra-high field MRI modalities, more specific delineations of these regions can facilitate greater understanding of mechanisms underlying stress-related function and psychopathology.

**Supplementary Information:**

The online version contains supplementary material available at 10.1007/s00429-023-02713-z.

## Introduction

Stress is a dynamic life factor that can influence neural circuitry, physiology and behavior; when the frequency and severity of stressors is regulated, learning and other behaviors might become more efficient (Levine 1971). However, when stress is not at an optimal level, one might experience maladaptive effects on the brain and body, including increased vulnerability to and/or manifestation of mental health disorders (Charney 2003). The high prevalence of mental health disorders, particularly anxiety and/or trauma-related disorders (Goldstein et al. 2016; Kessler et al. 2012; Koenen et al. 2017), warrants deeper examination of lesser explored nodes within stress responsive neural circuits, critical for further understanding the mechanisms underlying psychopathology risk and resilience.

The paraventricular nucleus of the hypothalamus (PVN) has arguably the most proximal neural control over the stress response, having access to both autonomic and neuroendocrine components (Herman et al. 2002). PVN subnuclei differentially innervate brainstem and spinal preganglionic neurons, controlling both parasympathetic and sympathetic divisions of the autonomic nervous system, respectively (Luiten et al. 1985). The PVN is also the gateway to the hypothalamic–pituitary–adrenal (HPA) axis, containing the corticotropin-releasing hormone (CRH) neurons that ultimately elicit glucocorticoid secretion (Herman and Cullinan 1997), and is a node of glucocorticoid negative feedback regulation (Herman and Tasker 2016; Reul and Kloet 1985). Thus, the PVN is critical for the function and regulation of physiological outcomes associated with stress responses.

The bed nucleus of the stria terminalis (BNST) is a heterogeneous limbic forebrain region with various subnuclei (Dong and Swanson 2003, 2004; Dong et al. 2001). Of note are the dorsal, oval nucleus, involved in preautonomic signaling, and the ventral, fusiform and anteroventral (fu/av) nuclei (Dong et al. 2001), which receive the densest viscerosensory, noradrenergic innervation in the brain (Aston-Jones et al. 1999; Fendt et al. 2005) primarily from the brainstem nucleus of the solitary tract and ventrolateral medulla (Aston-Jones et al. 1999; Delfs et al. 2000; Riche and DePommery 1990). Thus, the BNST is a central visceral nexus, integrating descending preautonomic and ascending viscerosensory signals.

In addition to its central visceral roles, the BNST controls/modulates physiological responses to stress via its direct connections to the PVN (Maita et al. 2021). Interestingly, the same ventral BNST subnuclei (fu/av) that receive viscerosensory input also directly and densely innervate the PVN (Dong et al. 2001), with the BNST-to-PVN projection stemming primarily from CRH neurons (Cullinan and Herman 1993; Herman et al. 1994). BNST subnuclei elicit differential control over PVN and physiological responses to stress (Crestani et al. 2013; Choi et al. 2004, 2007b), with the av BNST exerting inhibitory influence on stress-induced HPA responses (Johnson et al. 2016; Radley and Johnson 2018; Radley et al. 2009).

Extant research reflects heightened interest in the BNST, its role in fear and threat-related processes (Avery et al. 2016; Sullivan et al. 2004; Davis et al. 2010; Sink et al. 2013; Fox et al. 2008, 2018; Shackman et al. 2017), and resultant implications in anxiety (Davis et al. 2010; Walker and Davis 1997; Fendt et al. 2003) and trauma-related disorders (Sullivan et al. 2004; Gray and Piechowski 1993). Interestingly, the noradrenergic input, predominantly to the ventral BNST, may mediate conditioned and unconditioned fear leading to neurochemical rather than neuroanatomical hypotheses on functional dissociations (Schweimer et al. 2005).

The noradrenergic input to the ventral BNST also plays a role in stress-induced reinstatement of drug seeking (Leri et al. 2002), neural and behavioral responses to drug withdrawal (Aston-Jones et al. 1999; Delfs et al. 2000) and neuroendocrine and behavioral responses to psychological stress (Cecchi et al. 2002). Specific neurochemical lesions of this noradrenergic BNST-projecting pathway substantially attenuate anxiogenic, stress-induced corticosterone responses, indicating its importance in promoting stress responses (Banihashemi and Rinaman 2006). Importantly, this work also demonstrated that noradrenergic inputs to the BNST collateralize to provide most of the noradrenergic input to the CRH-rich, medial parvocellular PVN, indicating co-regulation of these structures by viscerosensory signaling. Despite the importance of this ventral, fu/av aspect of the BNST, a structural and functional delineation of the dorsal and ventral BNST in humans is currently lacking. For our purposes, oval and fu/av nuclei will be referred to in human brain as dorsal (dBNST) and ventral BNST (vBNST), respectively.

Due to their size and location in areas of potentially high signal dropout near ventricles, the PVN and BNST, and their relationship, have been less studied in humans. Recent work indicates BNST-PVN resting-state connectivity as a potential mediator between childhood trauma and affective disorders (Banihashemi et al. 2022). Further, BNST-hypothalamus structural connectivity may underlie sex differences in anxiety in the context of abstinence and Alcohol Use Disorder (Flook et al. 2021). Taken together, BNST, hypothalamus/PVN and their interactions are implicated in affective symptoms and stress-related psychopathology; further unpacking their specific anatomical relationships will facilitate greater mechanistic understanding of their contributions to these conditions.

Bridging the gap of region-specific influences on psychopathology may be improved with further parsing of the BNST and its subregion-specific relationship to the PVN. Our previous work utilized manual segmentations of PVN, and dorsal and ventral BNST (combined), on the 3 Tesla (T) ch2better template brain (Banihashemi et al. 2015, 2022; Wu et al. 2019). Avery et al. used a 7T gradient spin echo image to segment the dBNST (Avery et al. 2014). Torrisi et al. also went on to segment the dBNST at 7T (*n* = 27) (Torrisi et al. 2015), followed by Theiss et al.’s manual segmentation protocol (n = 10) and resultant dBNST probability map using T2-weighted imaging at 3 T (Theiss et al. 2017). At 3T, sufficient landmarks are present to accurately segment the dBNST, however, boundaries of the vBNST are less readily identifiable. Neudorfer et al. also segmented the BNST using one averaged 3T template; their primarily dorsal segmentation included a small portion of vBNST (Neudorfer et al. 2020). With the current work, we aimed to perform manual segmentation of our regions of interest (ROIs) at 7T, specifically using a multimodal approach utilizing T1-weighted MPRAGE and high resolution 2D gradient echo (GRE) in native space. Our use of the 7T GRE acquisition, in particular, provided visualization of anatomical landmarks that facilitated our novel identification of vBNST.

For the hypothalamus/PVN, previous approaches included whole hypothalamus segmentations using computer-assisted/semi-automated segmentation (Schindler et al. 2013; Wolff et al. 2018), broader parcellations [e.g., 5 parcels at 1.5T (Makris et al. 2013)], or subregions based on directionality or position (Spindler et al. 2020; Billot et al. 2020). Neudorfer et al. specifically segmented PVN (among other subnuclei) using one averaged 3T template (Neudorfer et al. 2020). Our approach builds on this foundational work with multiple native space 7T segmentations focused on isolating the PVN and preautonomic hypothalamus. With these segmentations of our ROIs (*n* = 25) using 7T acquisitions, we created probabilistic atlases in MNI space, determined inter-rater reliability (*n* = 10) and evaluated the alignment of our reverse-normalized probabilistic atlases with six additional native space manual segmentations.

Thus, the advantages and additional value of our approach include: (1) capitalizing on the greater signal-to-noise and resolution of native-space images at 7T to facilitate greater accuracy in identifying and isolating these structures; (2) utilizing GRE to enable visualization and identification of a new aspect of BNST, the vBNST; and (3) capturing and incorporating more individual neuroanatomical variability into the current probabilistic atlases by conducting multiple 7T segmentations in native space. The ultimate goal of this work is to provide additional resources to aid in further study of subregion-specific functions of, and interactions between the dBNST, vBNST and PVN. Investigating the dorsal–ventral distinction of the BNST in the context of the BNST–PVN relationship is a rich avenue for understanding the role of these circuits in emotion-related processes and mental health disorders.

## Materials and methods

### Participants

Participants were recruited from Allegheny County, Pittsburgh, PA, United States, utilizing various modalities (e.g., Pitt + Me research registry, Pittsburgh Regional Transit advertisements, and a variety of other online and print ads). In an ongoing study, an initial subsample of 31 participants was selected for manual segmentation procedures (25 were selected for manual segmentations, which contributed to probabilistic atlases, with an additional 6 participants for further evaluation of resultant probabilistic atlases). General exclusion criteria were: MRI or task-related contraindications (claustrophobia, ferrous metal in the body, body size, left-handedness, uncorrected visual impairments), pregnancy/breastfeeding, current bipolar disorder, history of psychotic disorders, past bipolar disorder with psychotic features, or current substance dependence.

Participants were young adults aged 20–35 years old (mean = 26.65, SD = 4.80); an adversity-enriched, transdiagnostic sample is being recruited, including individuals with mood, anxiety and/or trauma-related diagnoses, as determined by a trained interviewer using the Mini International Neuropsychiatric Interview (MINI) (Sheehan et al. 1998). Of the 25 participants whose segmentations were used for the probabilistic atlases, 17 were healthy (no history of affective diagnoses), 6 had past affective disorders [primary diagnoses: major depressive disorder (*n* = 4) and post-traumatic stress disorder (*n* = 2)] and 2 had current affective disorders (primary diagnoses: post-traumatic stress disorder). Participants with the highest quality MPRAGE and GRE images (i.e., minimal influence of noise, motion, and/or other artifacts) were prioritized for manual segmentation procedures. Informed consent was obtained from each participant. The current study was approved by the University of Pittsburgh Institutional Review Board.

### MRI acquisition

Scan slices were acquired using a 7T Magnetom Whole-Body MRI scanner (Siemens, Erlangen, Germany).

In this work, the 7T images were acquired using the first generation 16-ch Tx/32-ch Rx Tic Tac Toe RF coil system (Santini et al. 2018a, b, 2021; Krishnamurthy et al. 2019) on the single transmit mode.

A T1-weighted MPRAGE sequence was collected over 352 slices (voxel size = 0.55 mm isotropic, TE = 2.53 ms, TR = 3650 ms, and GeneRalized Autocalibrating Partial Parallel Acquisition (GRAPPA) acceleration factor = 2); the duration of the MPRAGE acquisition was 9 min and 56 s. Bias correction was performed by selecting the B1 filter (intensity medium); this is a native Siemens correction for this product sequence.

Coronal high-resolution images were acquired with 0.25 × 0.25 × 1.5 mm^3^ resolution using a 2D gradient echo (GRE) sequence (TR = 2980 ms, TE 1 = 14 ms and TE 2 = 28 ms (TE 1 was used for segmentations); FoV = 205 mm (HF)  ×  167 mm (RL), distance factor = 20%, and GRAPPA acceleration factor = 3. Sixty-seven slices were collected; the duration of this acquisition was 9 min and 40 s. In the sequence parameters, we selected the option “normalize” (2D, medium intensity) to bias correct the images; this is a native Siemens correction for this product sequence.

The GRE scans were coregistered, using SPM12’s ‘Coregister: Estimate and Reslice’ option, to the participant’s respective MPRAGE for the dBNST and vBNST segmentation processes (Fig. S1). We collected approximately 1 h of scanning data (other modalities not shown).

### Segmentations

Segmentations for 25 participants were conducted by BMS (Rater 1) in native space, who was the expert rater (trained by LB). For reliability purposes, ten duplicate MPRAGE images were subsequently segmented by DL (Rater 2). In addition, six new segmentations (three done by each rater) were completed for all three regions to evaluate the efficacy of our probabilistic atlases. Three of the six participants had some psychiatric history, however, none had any current psychopathology. Primary diagnoses were major depressive disorder, PTSD, and panic disorder.

#### BNST segmentation

**Fig. 1:**
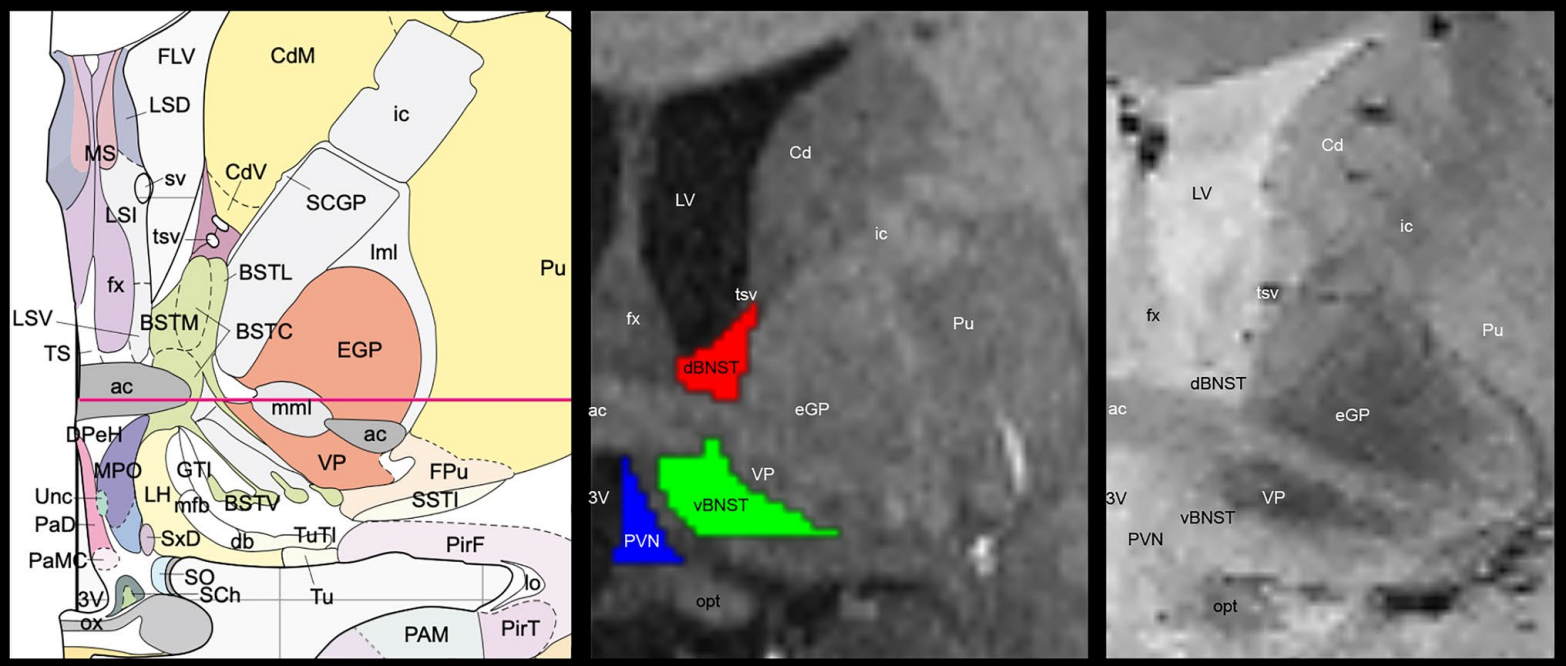
The paraventricular nucleus of the hypothalamus (PVN) and the bed nucleus of the stria terminalis (BNST) and their surrounding boundary-setting structures; tract. Left = Atlas of the Human Brain (Mai et al. 2008; ‘BST’ structures used to depict dorsal and ventral BNST and PaD (paraventricular nucleus, dorsal part) and PaMC (paraventricular nucleus, magnocellular part) used to depict PVN structure); Middle = T1-weighted MPRAGE with segmentations (dorsal BNST, red; ventral BNST, green; PVN, blue); Right = high-resolution gradient echo (2D GRE). *dBNST* dorsal bed nucleus of stria terminalis, *vBNST* ventral bed nucleus of stria terminalis, *PVN* paraventricular nucleus of the hypothalamus, *ac* anterior commissure, *Cd* caudate, *eGP* external globus pallidus, *ic* internal capsule, *LV* lateral ventricles, *tsv* thalamostriate vein, *VP* ventral pallidum, *Pu* putamen, *3V* third ventricle, *opt* optic tract

Both MPRAGE and GRE modalities were used to define the dBNST and vBNST. Using SPM12’s display function, the anterior commissure was identified as the origin for both MPRAGE and GRE, then aligned to the AC–PC plane to aid coregistration. Each participant’s GRE was coregistered to their MPRAGE image and were kept in native space for segmentation. Segmentations were completed manually on coronal sections using MRIcroGL’s draw function. The decussation of the anterior commissure was identified first to locate the starting point for segmentations; then raters would work anteriorly and posteriorly from that slice to the point where the anterior commissure does not overlap with the ventral pallidal area and the fornix separates, respectively. Segmentations were drawn slice by slice, ensuring that the dorsal and ventral aspects of the BNST were confined within the boundaries, as seen in slices 18–24 in the Atlas of the Human Brain (Mai et al. 2008), which was used as a reference template for the segmentation process. The boundaries included the anterior commissure, lateral ventricles, fornix, thalamostriate vein, internal capsule and ventral pallidal area, each with varying prominence depending on the slice. The dBNST was defined as the area confined by the boundaries above the anterior commissure, similar to the approach of Avery et al. (2014), Theiss et al. (2017), whereas the vBNST was defined as the area within the boundaries below the anterior commissure (Fig. 1).

Segmentations were then refined using an “angular approach” process (Fig. S1). The angular approach was formulated by opening the atlas images via GNU Image Manipulation Program (GIMP) to measure the angle from the center of the anterior commissure to the superior- and inferomedial-most and superior- and inferolateral-most portions of the dBNST and vBNST for every slice in which they were visible. Once these angles were measured out on the atlas, and the segmentation was complete in native space, each slice of the segmentation was opened in GIMP, where the angle was measured again to match with the atlas measurement. If the boundaries were clearly visible, the angular measurements were consulted but bore less weight, as they were not necessary for boundary identification. Thus, this process was most instructive where boundaries were difficult to distinguish (e.g., signal dropout near the pallidal area, difficulty identifying thalamostriate vein).

#### PVN segmentation

Only the MPRAGE modality was utilized to identify and segment the PVN, due to some signal dropout surrounding the third ventricle in the GRE. PVN segmentations were performed after BNST segmentations, so images were already in proper alignment (see steps above). PVN segmentations were drawn on coronal slices with the BNST segmentations visible, so that there would not be any overlap between the regions. The PVN was anteriorly defined by where the third ventricle began to take shape, being tucked between the third ventricle (medially) and the nucleus of the diagonal band (laterally). The PVN was also bound by the anteroventral periventricular hypothalamic nucleus (inferior), and by the diagonal band (superiorly). Working posteriorly, the PVN segmentation maintained a triangular shape, with its size growing, respectively, with the boundaries as they shifted through the slices (Fig. 1). When the anterior commissure decussates, the PVN is bounded laterally by the lateral hypothalamic area, medially by the third ventricle, superiorly by the anterior commissure, and inferiorly by the suprachiasmatic nucleus. These boundaries are upheld as the anterior commissure dissipates laterally and the fornix becomes more prominent. Once the fornix separates, the PVN begins to extend superiorly, tucking up and to the side of the fornix, bound medially by third ventricle, laterally by the fornix, superiorly by the fornix and/or third ventricle, and inferiorly by the ventromedial hypothalamic nucleus. The segmentation concluded posteriorly once the fornix fully separated, as the posterior-most PVN was not identifiable tucked among several other nuclei.

### Generating BNST and PVN probabilistic atlases

For each of the 25 original segmentations, we conducted normalization to a standard anatomic space (the Montreal Neurological Institute (MNI) space). We used Statistical Parametric Mapping (SPM12) (Penny et al. 2011) in MATLAB 2021b (MathWorks, Natick, MA, USA) to segment the MPRAGE into six tissues classes (Ashburner and Friston 2005). This process generates deformation fields, which can be used to normalize images that are coregistered to the MPRAGE, to MNI space. This deformation field was applied to all three segmentations (i.e., dBNST, vBNST and PVN) for each participant. We then computed the probability that each voxel in MNI space was a part of each region (i.e., voxels with a value of 1 indicate that all participants included this voxel as part of the segmentation). This was done for each region separately.

### Inter-rater reliability and probabilistic atlas/segmentation reliability

Inter-rater reliability was assessed using the Dice similarity coefficient, as it provides insight into the overlap of the voxel-based segmentations. Calculations for inter-rater reliability were separate for vBNST, dBNST and PVN. Dice coefficients can be interpreted as low (0.00–0.19), low-moderate (0.20–0.39), moderate (0.40–0.59), moderate-high (0.60–0.79) or high (0.80–1.00) (Elin et al. 2022; Wilson et al. 2017).

For the six participants who were not included in the probabilistic atlas generation, we reverse normalized (using the inverse deformation field) each of the probabilistic atlases. For each region, we then computed Dice coefficients as a function of probability threshold (0.1–1) between the now native space probabilistic atlases and the manual segmentations. This is to evaluate how well the templates map *back* onto native space segmentations.

## Results

**Fig. 2:**
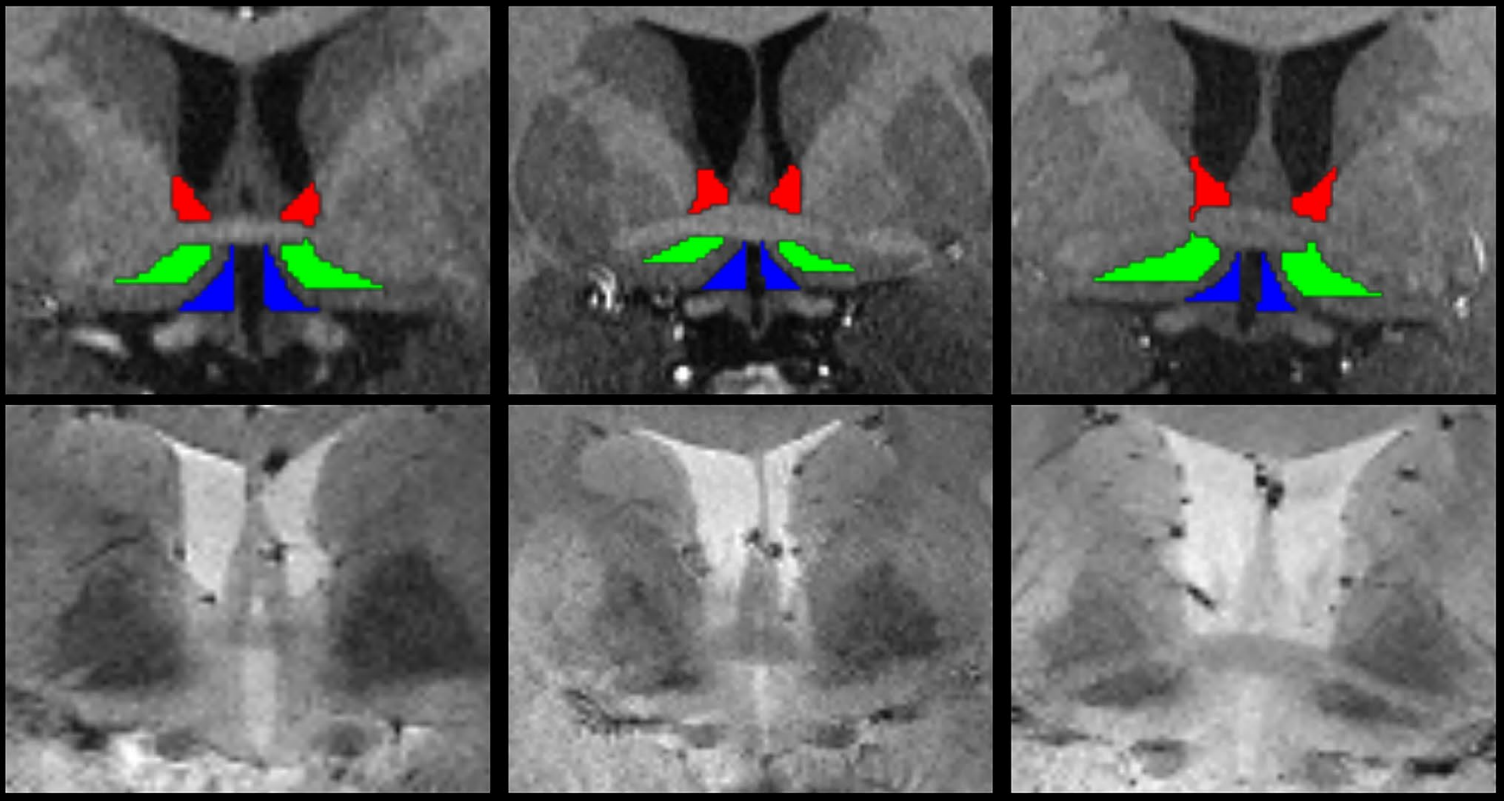
Manual segmentations of the dorsal (red) and ventral (green) bed nucleus of the stria terminalis and paraventricular nucleus of the hypothalamus (blue) in three representative native space T1-weighted MPRAGE images (Top) with respective high resolution gradient echo (2D GRE) (Bottom), demonstrating how each segmentation is uniquely influenced by individual neuroanatomical differences in boundary-driving structures

See Supplemental Information for segmentation characteristics (Table S1, *n* = 31). See Fig. 2 for individual differences in the neuroanatomical characteristics contributing to variability of the native space segmentations.

For the ten participants that were rated by both raters, we found moderate-high inter-rater reliability across regions (Wilson et al. 2017) (Dice Coefficient Mean (SD); PVN = 0.69 (0.04); dBNST = 0.77 (0.04); vBNST = 0.62 (0.04)).

**Fig. 3:**
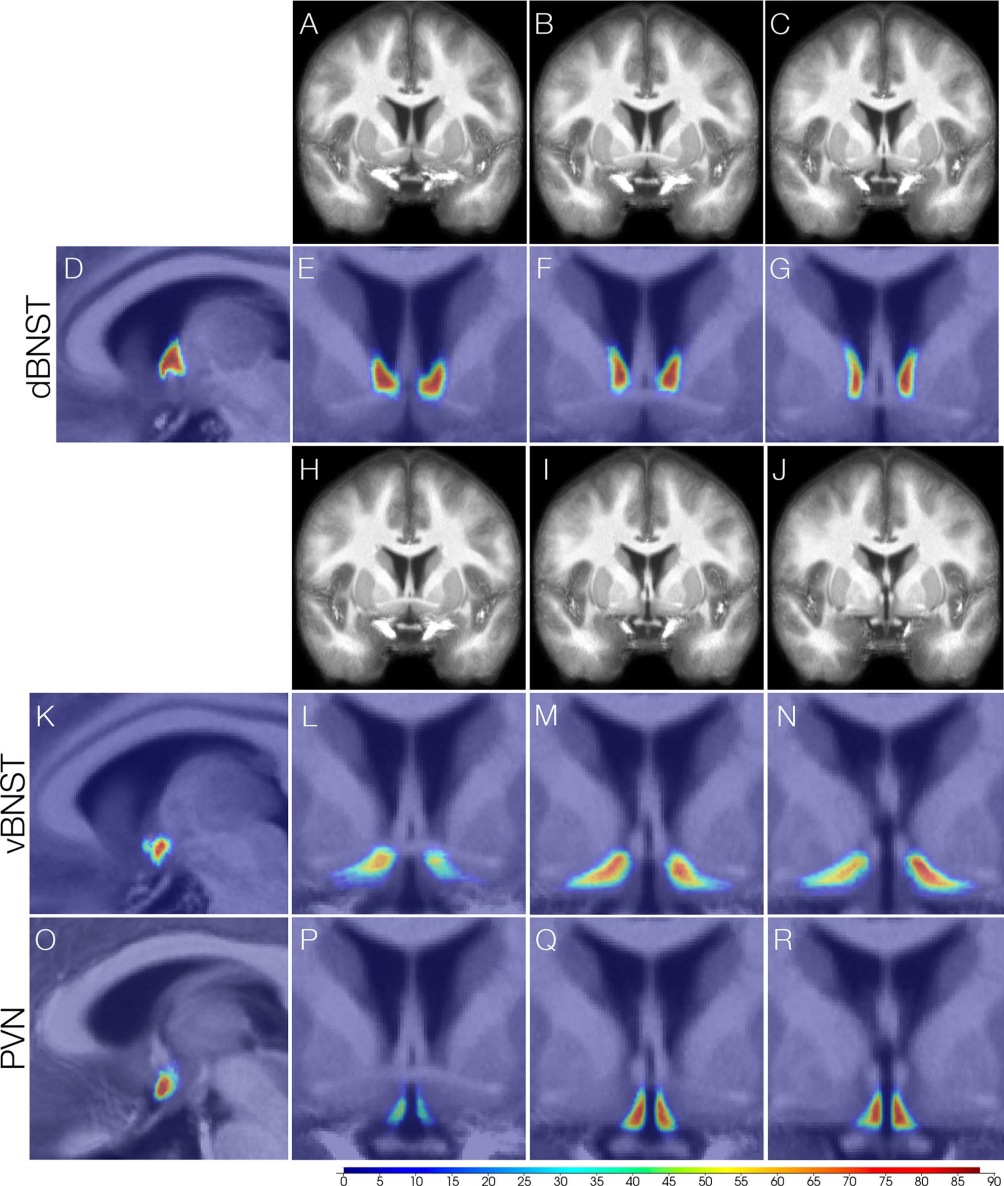
Probabilistic Atlases for dorsal BNST (dBNST), ventral BNST (vBNST) and PVN. **A**–**C** Coronal slices of the averaged MPRAGE (*n* = 25) across three anterior–posterior sections with (**D**, sagittal view of dBNST probabilistic atlas) corresponding dorsal BNST probabilistic atlas depictions (**E**–**G**). **H**–**J** Coronal slices of the averaged MPRAGE across three anterior–posterior sections with (**K**, sagittal view of vBNST probabilistic atlas) corresponding ventral BNST probabilistic atlas depictions (**L**–**N**) and (**O**, sagittal view of PVN probabilistic atlas) corresponding PVN probabilistic atlas depictions (**P**–**R**)

The template probabilistic atlases for each of the three regions (see Table 1 for characteristics) are overlaid onto a MNI space MPRAGE image averaged across the 25 participants (Fig. 3). The probabilistic atlases and this average MPRAGE image are now shared resources as part of this manuscript (Supplemental files).

**Table 1:** Probabilistic Atlas characteristics (n = 25)

Probabilistic Atlas	Center of Mass (MNI space coordinates)	Min (voxels)	Mean (voxels)	Max (voxels)	Standard Deviation	Volume (mm^3^)
dBNST	0.05, 2.83, – 0.53	46.33	129.64	192.36	32.30	950
vBNST	0.979, 0.27, – 8.65	113.45	157.41	195.49	15.61	1430
PVN	0.46, – 0.66, – 10.90	50.06	114.13	183.55	27.00	590

Characteristics for probabilistic atlases, with min, mean and max referring to the number of voxels in a given slice

**Fig. 4:**
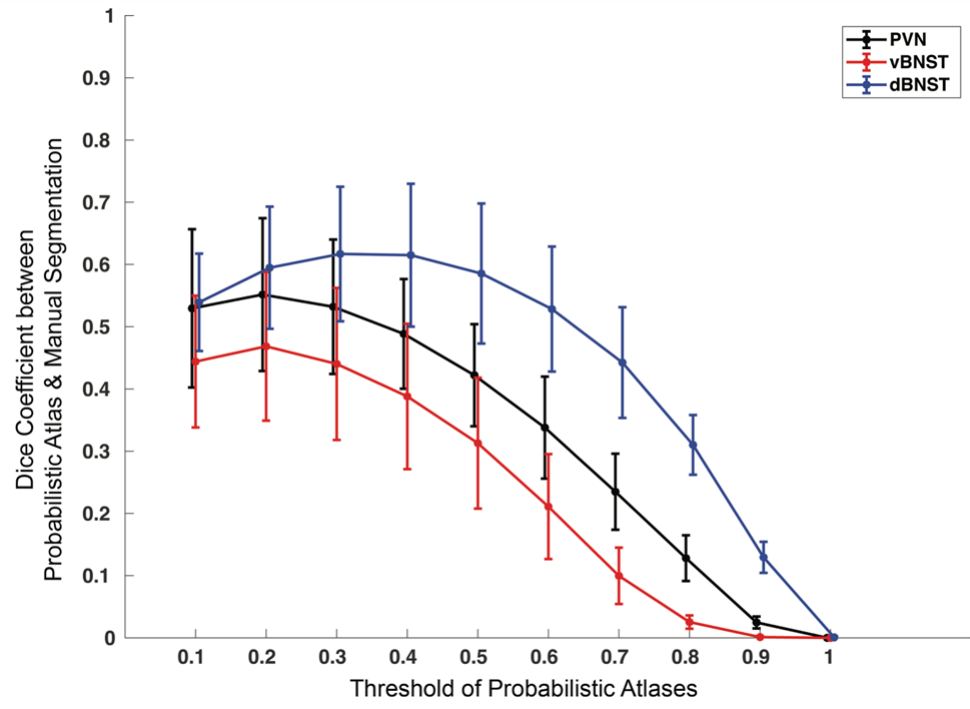
Dice coefficients between probabilistic atlases and manual segmentations as a function of threshold (*n* = 6). For each region, Dice coefficients were computed as a function of probability threshold (0.1–1) between the reverse transformed native space probabilistic atlases and the manual segmentations in order to evaluate how well the templates map *back* onto native space segmentations

In the six participants who were not included in the segmentations used for the generation of the probabilistic atlases, we reverse normalized these probabilistic atlases and evaluated the Dice coefficients as a function of threshold (Fig. 4). The best Dice coefficients were generated at a threshold of 0.2, where we found moderate to moderate–high agreement between the probabilistic atlases and the manual segmentations [Dice Coefficient Mean (SD); PVN = 0.55 (0.12); dBNST = 0.60 (0.10); vBNST = 0.47 (0.12 SD)].

## Discussion

The current study utilized 7T imaging to manually segment the BNST and PVN and was unique in identifying and segmenting the dorsal and ventral BNST with guidance from high-resolution GRE data and coregistered MPRAGE images. Further, our segmentation protocol demonstrated moderate–high reliability across raters and allowed for the construction of probabilistic atlases, providing a robust method of identifying the PVN and delineating the dorsal and ventral BNST. These probabilistic atlases were also reverse normalized to native space and showed moderate-to-moderate–high reliability in six participants not included in the original atlases. These probabilistic atlases are now shared with the wider neuroimaging community, along with the averaged template brain (*n* = 25).

The relationship between the BNST and the PVN, as well as their function within a central visceral network (Banihashemi et al. 2022; Rinaman et al. 2011), has been shown to be critical for the control of physiological stress responses (Choi et al. 2007a, b; Crestani et al. 2013). Building on preclinical work indicating early experience-related structural and functional alterations in PVN and BNST (Banihashemi and Rinaman 2010; Banihashemi et al. 2011; Card et al. 2005), work in healthy adults showed that greater childhood physical abuse was associated with greater BNST and PVN stressor-evoked activity (Banihashemi et al. 2015).

In investigating functional dynamics between BNST and PVN, our recent work showed that greater childhood threat, namely traumatic experiences, was associated with lower BNST-PVN resting-state connectivity in an abuse-enriched, transdiagnostic sample (Banihashemi et al. 2022). This work also demonstrated that greater BNST–PVN resting-state connectivity was associated with fewer lifetime affective diagnoses, implicating this neural connection as a potential mediator between childhood threat and affective vulnerability (Banihashemi et al. 2022). While childhood adversity has been associated with aberrant connectivity and structural changes in limbic regions, such as the amygdala and prefrontal cortex (Hart and Rubia 2012), the influence of the BNST, PVN and their connectivity is understudied in this context and presents potentially important mechanisms by which stress-related neural alterations contribute to psychopathology development.

To this end, our ongoing study is recruiting a childhood abuse-enriched, transdiagnostic sample. This sample is recruiting a full distribution across childhood abuse severity classifications (none–minimal, low–moderate, moderate–severe, severe–extreme). These participants include healthy individuals and those with a range of affective symptoms, including those that meet threshold for clinical affective disorders. Thus, our goal is to capture and include individual differences in neuroanatomy, which may include variations related to psychopathology. However, for the current segmentations contributing to the probabilistic atlases, we aimed for a predominantly healthy sample. Further, exploratory analyses revealed no between-group (healthy, past psychopathology, current psychopathology) differences in dBNST (*p* = 0.11), vBNST (*p* = 0.14) or PVN (*p* = 0.07) volume (see Supplement, Table S2).

For the manual segmentations and probabilistic atlas development, our approach used a comparable and, in some cases, larger sample size for BNST or hypothalamus/PVN (Table S3) and is unique in utilizing 7T GRE to visualize and segment vBNST. An exploratory analysis revealed that the net benefit of adding participants to an average template for the dBNST decreases rapidly after just a few participants, suggesting that 25 participants is a reasonably robust sample size for manual segmentations (See Supplement, Evaluation of Sample Size (n = 25) for Probabilistic Atlas Generation, Fig. S2). For dBNST, we achieved similar inter-rater reliability to that of Theiss et al. (Theiss et al. 2017) and a more robust inter-rater reliability than that of Torrisi et al. (Torrisi et al. 2015), however, all were in the moderate-high range. For PVN, our inter-rater reliability was more modest (moderate–high) compared to that reported by Neudorfer et al. (high) (Neudorfer et al. 2020); however, the comparison is not equivalent (i.e., two raters in one template brain vs. ten manual segmentations in native space.) (See Supplement, Comparisons with Previous Approaches; Fig. S3.)

A limitation of the present work is the challenge of identifying these structures in the context of individual differences in neuroanatomical features of each native space brain. Some images contained structures, such as the thalamostriate vein, that were difficult to identify due to their size and location relative to dropout-prone regions (e.g., lateral ventricles), which made identifying the superior extent of the dBNST more challenging. Similarly, some individuals displayed an anterior commissure or fornix that was prominent for one or two slices before separating, whereas other individuals had these structures intact for three to four slices. For posterior-most slices, in which the anterior commissure and fornix were already separated, it was difficult to identify more medial components of both segmentations, especially the PVN, given the lack of distinction between it and other small local nuclei. In aforementioned slices, the space between fornix and third ventricle was too compact at times to definitively assign voxels to a segmentation, such that there was no overlap between the segmentation and the ventricle or fornix. These differences made it difficult to segment based on slice-specific orientations of structures as seen in the Atlas of the Human Brain (Mai et al. 2008), however, this was mainly combatted with our angular approach (Fig. S1). Due to their proximity to one another, the angular approach used on the BNST enabled more accuracy and precision in honing in on the PVN across individuals compared to broader approaches based on directionality or position (Spindler et al. 2020; Billot et al. 2020). Further, native space segmentations may capture more of the natural neuroanatomical variability between individuals that may otherwise be missed in template-based atlases (Neudorfer et al. 2020).

Another limitation was that PVN segmentations had only one usable imaging modality, unlike the two options afforded to the BNST (i.e., MPRAGE and GRE). Further, due to its size, the medial preoptic nucleus was partially incorporated into the segmentation, as it was not easily identifiable. However, in tandem with the angular approach implemented for BNST and the boundary of our BNST segmentation itself, we created a line from the medial side of the fornix to the lateral aspect of the optic tract as a boundary to limit the inclusion of regions like the medial preoptic nucleus. Similarly, given that the extent of the vBNST was thin and could be difficult to discern, segmentations included areas interspersed with the great terminal island, however, previous work indicates functional signal is present throughout this region (Banihashemi et al. 2015). Lastly, the current study only evaluated the probabilistic atlases for 7T images; future work is required in applying these probabilistic atlases to 3T images.

## Conclusion

In this study, manual segmentations of the PVN and dorsal and ventral BNST were performed using 7T neuroimaging. Multimodal neuroimaging was used to delineate dorsal and ventral BNST subregions. We demonstrated moderate-high inter-rater reliability, and good overlap between the reverse transformed probabilistic atlases and additional manual segmentations. Future directions can include assessments of gray matter volumes of these regions in various developmental, social and clinical contexts, as well as ROI-to-ROI or ROI-whole brain connectivity analyses to investigate functional connectivity within large-scale networks. With the current probabilistic atlases, future studies can delineate the distinct functions of the PVN and BNST’s dorsal and ventral subregions to provide insights into subregion-specific contributions to dysregulation of stress responses, anxiety, addiction and other stress-related psychopathologies.

### Supplementary Information

Below is the link to the electronic supplementary material.

Supplementary file1 (PDF 5167 KB)

Supplementary file2 (PDF 1769 KB)

Supplementary file3 (NII 255523 KB)

Supplementary file4 (NII 41750 KB)

Supplementary file5 (NII 255523 KB)

Supplementary file6 (NII 255524 KB)

## Data Availability

The averaged MPRAGE template (*n* = 25) and the probabilistic atlases for dorsal BNST, ventral BNST and PVN are now available as Supplemental files and publicly available (https://www.brainbodystress.pitt.edu).
