# Supplementary material for: Manual segmentation of the paraventricular nucleus of the hypothalamus and the dorsal and ventral bed nucleus of stria terminalis using multimodal 7 Tesla structural MRI: probabilistic atlases for a stress-control triad
Source: Brain Struct Funct. 2023 Oct 9;229(2):273–83. doi: 10.1007/s00429-023-02713-z (PMC10917873; doi:10.1007/s00429-023-02713-z)
Supplement: Supplementary file 1 — Supplementary file1 (PDF 5167 KB) [file 429_2023_2713_MOESM1_ESM.pdf]

Manual Segmentation of the Paraventricular Nucleus of the Hypothalamus and the Dorsal and Ventral Bed Nucleus of Stria Terminalis using Multimodal 7 Tesla Structural MRI: Probabilistic Atlases for a Stress-Control Triad

Brandon M. Sibbach[1], Helmet T. Karim[1,2], Daniel Lo[1], Nithya Kasibhatla[1], Tales Santini[2], Jessica C. Weber[1], Tamer S. Ibrahim[2], Layla Banihashemi[1,2]

[1]Department of Psychiatry, University of Pittsburgh, Pittsburgh, PA 15213

[2]Department of Bioengineering, University of Pittsburgh, Pittsburgh, PA 15213

Corresponding author:

Layla Banihashemi, Ph.D.

Layla.banihashemi@pitt.edu

https://orcid.org/0000-0001-6317-0494

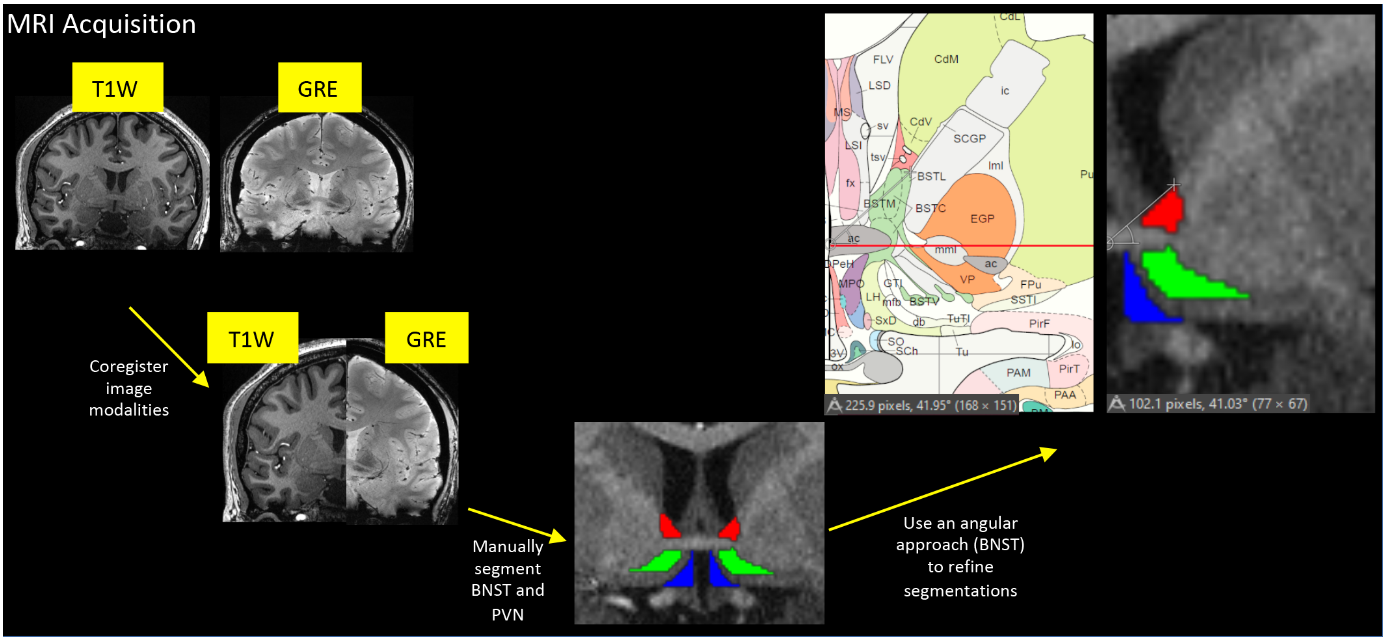

Figure S1. Schematic of manual segmentation protocol from image acquisition to segmentation refinement. T1-weighted (T1W) MPRAGE and Gradient Echo (GRE) modalities were utilized for dorsal (red) and ventral BNST (green) segmentations. MPRAGE only was used for PVN segmentations (blue).

Table S1. Segmentation Characteristics (n=31)

Segmentation	Voxels Mean (SD)		Volume (mm^3) Mean (SD)	
dBNST	1201.96	(231.61)	203.57	(33.02)
vBNST	1346.68	(350.62)	228.57	(51.48)
PVN	636.96	(181.66)	107.86	(27.13)

Dorsal BNST (dBNST), ventral BNST (vBNST) and paraventricular nucleus of the hypothalamus (PVN)

Evaluation of Mean Volume Differences Based on Sample Composition

We aimed for a sample of predominantly healthy individuals for the 25 manual segmentations. ANOVA revealed no between-group differences in dBNST (p = 0.11), vBNST (p = 0.14) or PVN (p = .07) volume (Table S2).

Table S2. Mean ROI Volumes Based on Psychopathology

		Volume (cubic cm)		
		dBNST	vBNST	PVN
Healthy (n=17)	Mean	.211	.214	.098
	SD	.037	.047	.023
Psychopathology (Current or Past) (n=8)	Mean	.231	.245	.120
	SD	.042	.048	.030
	p	.237	.142	.049
	B-H adj *p*	.237	.213	.147

*B-H - Benjamini-Hochberg adjusted P value

Table S3. Comparison of Selected BNST and Hypothalamic Segmentation Approaches

	Current Study	(Avery et al. 2014)	(Theiss et al. 2017)	(Schindler et al. 2013)	(Neudorfer et al. 2020)	(Spindler et al. 2020)	(Billot et al. 2020)
Sample Size	n = 25	n = 1	n = 10	n = 10	n = 1 template	n = 98	n = 37
Interrater Reliability	n = 10	n/a (two raters confirmed a single rater's segmentation)	n = 10	n = 10	n = 2; 1 template	n = 29	n = 10
Approach	Native Space	Native Space	Native Space	Algorithm	Template	Native Space	Native Space
Field Strength	7-Tesla	7-Tesla	3-Tesla	7-Tesla	3-Tesla	3-Tesla	3-Tesla
Included ROIs	Dorsal BNST, Ventral BNST, PVN	Dorsal BNST	Dorsal BNST	Entire hypothalamus (nuclei were not parsed)	PVN (as well as other hypothalamic areas) & BNST (dorsal and partial ventral combined)	Hypothalamus delineated by directionality (anterior-superior, anterior-inferior, intermediate, and posterior hypothalamus)	Hypothalamic subunits (anterior-superior, anterior-inferior, superior-tuberal, inferior-tuberal, and posterior hypothalamus)
Image Modalities	T1-Weighted MPRAGE (T1W)<br><br>High-resolution Gradient Echo (GRE)	Gradient Spin Echo (GRASE)	T2-Weighted Turbo Spin Echo (TSE)<br><br>T1-Weighted MPRAGE	T1-Weighted MP2RAGE	T1-Weighted MPRAGE<br><br>T2-Weighted MPRAGE	T1-Weighted MPRAGE<br><br>T2-Weighted MPRAGE	T1-Weighted MPRAGE<br><br>T2-Weighted MPRAGE

Evaluation of Sample Size (n=25) for Probabilistic Atlas Generation

We evaluated the net benefit of adding participants to an average template for the dBNST one at a time. We first identified two participants whose segmentation had the greatest Dice coefficient. We then computed a template from these two participants and recomputed the Dice between this new template and all other participants. We then iteratively repeated this process, each time computing the Dice between the previous template and the current participant with the greatest Dice. We show that the Dice coefficient drops from ~0.7 to ~0.62-0.64 after just a few participants (see Fig. S2). This shows that adding successive participants does significantly change the template; while there is noise around which participant is added, largely these templates 'converge' to an average relatively quickly.

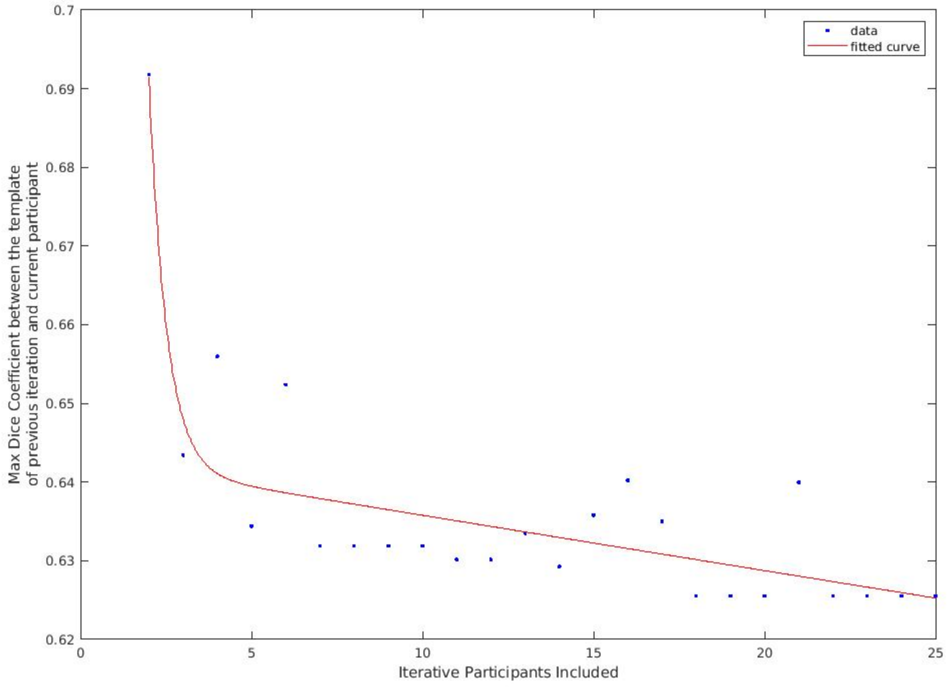

Figure S2. Evaluation of Sample Size. Plots shows maximum Dice coefficient between the template of previous iteration and current participant (y-axis) and the iterative participants included (x-axis). The Dice coefficient drops from ~0.7 to ~0.62-0.64 after just a few participants.

Comparisons with Previous Approaches

In order to evaluate the alignment and/or overlap of our atlas with those of previous work, we conducted an analysis using the Blackford dBNST probabilistic atlas (Theiss et al. 2017). Using the 10% Blackford atlas (reported as having the best overlap with their 7T mask (Avery et al. 2014)) and our 20% dBNST atlas, we found a Dice of 0.84. Thus, these are largely overlapping, differing somewhat at the tails and outer boundaries (Fig. S3). An analysis comparing the Neudorfer PVN mask (Neudorfer et al. 2020) with our 20% PVN probabilistic atlas revealed a Dice of 0.34. These differed overall in size and shape, with our current atlas being larger and including more ventral areas.

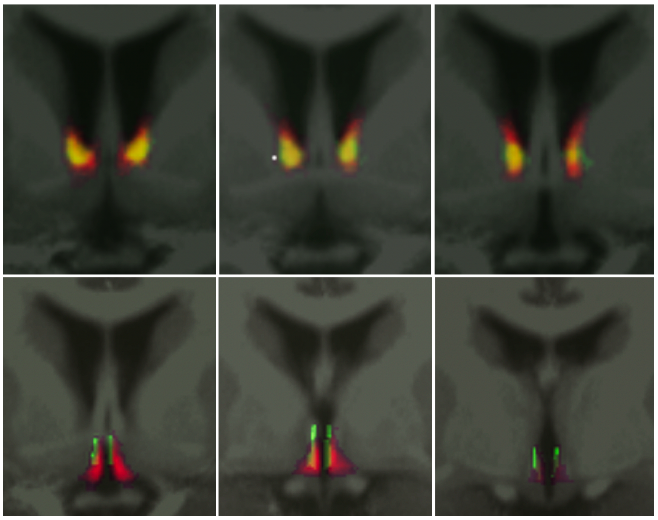

Figure S3. Comparisons with Previous Work. Top – Three images through the BNST demonstrating the overlap between the full Blackford BNST probabilistic atlas (green) and the current full dBNST probabilistic atlas (red). Bottom - Three images through the PVN demonstrating the overlap between the Neudorfer mask (green) and the current full PVN probabilistic atlas (red).

For dBNST, our inter-rater reliability (Dice = 0.77) was similar to that of Theiss et al. who reported their Dice as 0.79 (left) and 0.77 (right) (Theiss et al. 2017), and more robust than that of Torrisi et al. who reported their Dice as 0.61 for both left and right BNSTs, however, these were all in the moderate-high range. The Neudorfer BNST segmentation is primarily “dorsal” compared to our segmentations, however, included a

relatively small portion our "ventral" BNST. The Neudorfer BNST does not include the portion of the ventral BNST that extends under the pallidal area. Neudorfer et al. reported their mean Dice as 0.497 (Neudorfer et al. 2020), which is considered moderate, while our inter-rater reliability for dBNST (Dice = 0.77) and vBNST (Dice = 0.62) were both moderate-high.

For PVN, Neudorfer reported their mean Dice for hypothalamic nuclei as 0.63, while their mean Dice for PVN ('Pa') was 0.92 (Neudorfer et al. 2020); our inter-rater reliability for PVN was more modest, a Dice of 0.69 (moderate-high), however, this is not an equivalent comparison due the Neudorfer inter-rater reliability being based on two raters' segmentations in one template brain, while ours is based on 10 native space segmentations.

References

Avery SN, Clauss JA, Winder DG, Woodward N, Heckers S, Blackford JU (2014) BNST neurocircuitry in humans. NeuroImage 91:311-323. doi:https://doi.org/10.1016/j.neuroimage.2014.01.017

Billot B, Bocchetta M, Todd E, Dalca AV, Rohrer JD, Iglesias JE (2020) Automated segmentation of the hypothalamus and associated subunits in brain MRI. NeuroImage 223:117287. doi:10.1016/j.neuroimage.2020.117287 PMID - 32853816

Neudorfer C, Germann J, Elias GJB, Gramer R, Boutet A, Lozano AM (2020) A high-resolution in vivo magnetic resonance imaging atlas of the human hypothalamic region. Sci Data 7 (1):305. doi:10.1038/s41597-020-00644-6 PMID - 32934244

Schindler S, Schönknecht P, Schmidt L, Anwander A, Strauß M, Trampel R, Bazin P-L, Möller HE, Hegerl U, Turner R, Geyer S (2013) Development and Evaluation of an Algorithm for the Computer-Assisted Segmentation of the Human Hypothalamus on 7-Tesla Magnetic Resonance Images. PLoS ONE 8 (7):e66394. doi:10.1371/journal.pone.0066394 PMID - 23935821

Spindler M, Özyurt J, Thiel CM (2020) Automated diffusion-based parcellation of the hypothalamus reveals subunit-specific associations with obesity. Scientific Reports 10 (1):22238. doi:10.1038/s41598-020-79289-9 PMID - 33335266

Theiss JD, Ridgewell C, McHugo M, Heckers S, Blackford JU (2017) Manual segmentation of the human bed nucleus of the stria terminalis using 3T MRI. NeuroImage 146 (C):288 - 292. doi:10.1016/j.neuroimage.2016.11.047
