# Supplementary material for: Manual segmentation of the paraventricular nucleus of the hypothalamus and the dorsal and ventral bed nucleus of stria terminalis using multimodal 7 Tesla structural MRI: probabilistic atlases for a stress-control triad
Source: Brain Struct Funct. 2023 Oct 9;229(2):273–83. doi: 10.1007/s00429-023-02713-z (PMC10917873; doi:10.1007/s00429-023-02713-z)

SPM Reorientation Protocol (run in MATLAB via ‘spm fmri’):

1. In the SPM GUI, select Display, then navigate to the appropriate folder containing the MPRAGE and GRE images to be segmented over

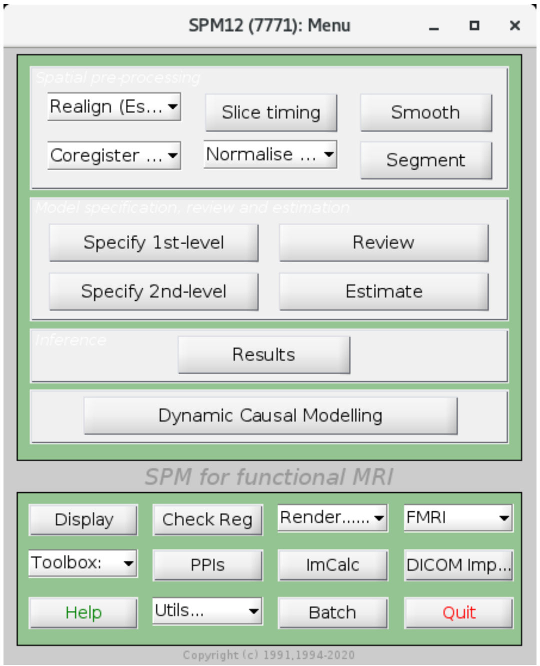

2. Open the MPRAGE, then move the blue crosshairs to locate the anterior commissure

3. Maintaining the position of the crosshairs, adjust the pitch, roll, and yaw of the image to align the anterior commissure and the posterior commissure (AC-PC alignment)

4. Click ‘Set Origin’, then ‘Reorient’ (move the Crosshair off the AC, then click ‘Origin’ to ensure the origin was properly set)

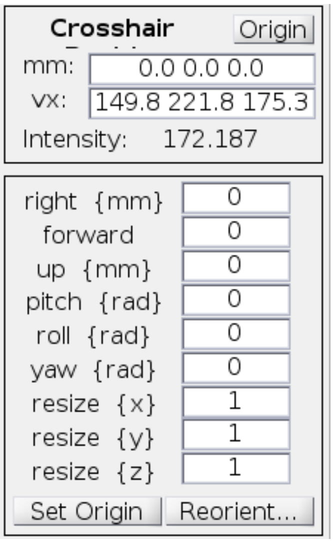

5. Save the file (repeat steps for GRE)

SPM Coregistration Protocol (run in MATLAB via ‘spm fmri’):

1. In the SPM GUI, select ‘Coregister: Estimate and Reslice”, then navigate to and select the appropriate MPRAGE (reference image) and GRE (source image)

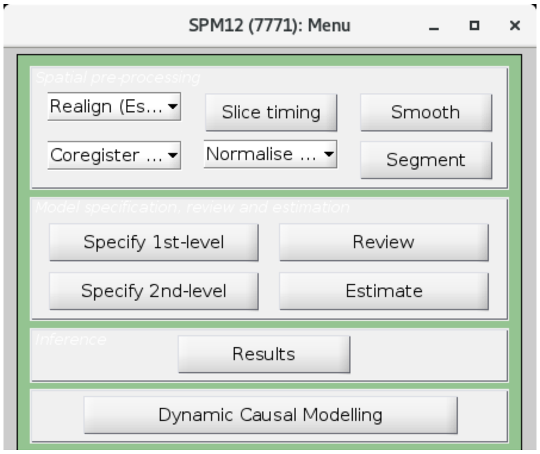

2. Navigate to and select the appropriate MPRAGE (reference image) and GRE (source image). Leave all other options as they are, and select the 'run' icon

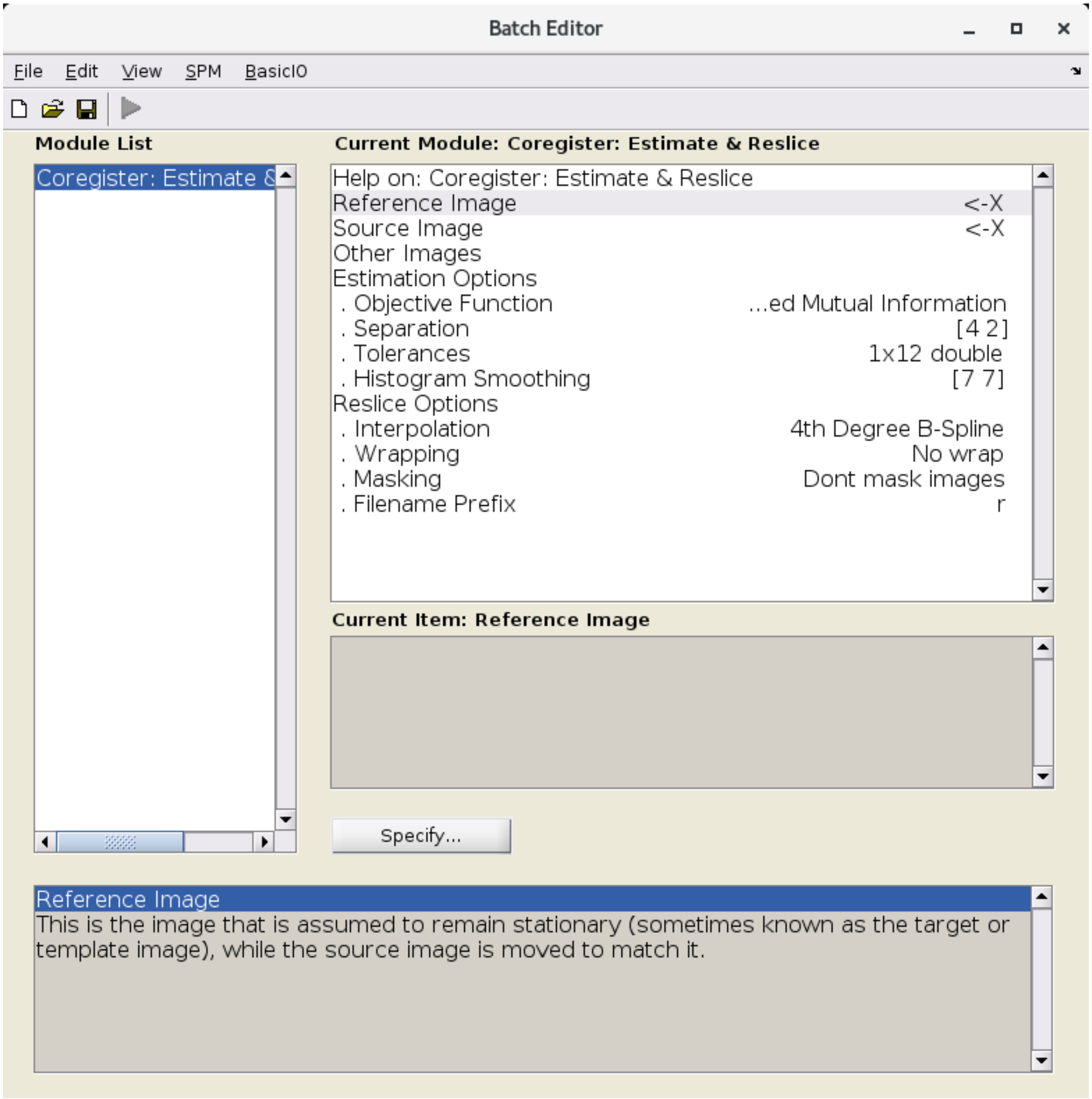

3. Ensure both images are properly aligned in the 'Display' option, or by overlaying images in MRIcroGL or another image viewing program
4. Proceed to ROI segmentation protocol

<u>ROI Segmentation:</u>

Software/References needed: MRIcroGL; GIMP (GNU Image Manipulation Program); Mai et al. atlas PDF

Notes:

- <u>Draw tool</u>:
  - to activate, click *Draw* → *Draw Color* → *Red,* or "CTRL 1"
  - Different colors are used to denote either different ROIs, or different portions of one ROI (i.e., for the BNST, red will denote the dorsal portion, while green denotes the ventral extent; PVN will be blue)
  - The draw tool can be finicky and loves to autofill your drawing for you, sometimes for the worse
    - To ensure the autofill happens within the area you want, draw from a start point and make sure you land back at that point before releasing your cursor
    - This goes the same for when you are erasing to refine your drawing. Even if you are erasing one or two voxels, make sure to circle back to the point you started at (i.e., erase the voxel plus a circle of an area that is not drawn on)
    - Sometimes it will still happen, so just use "CTRL U" to undo that mistake and try again!
- <u>Image navigation:</u>
  - Scrolling up moves you anteriorly, scrolling down moves you posteriorly
  - Holding 'Ctrl' while left clicking allows you to drag the entire image (including your segmentation)
  - Holding 'Ctrl' while scrolling allows you to zoom in and out

Step 1: Open coregistered images (MPRAGE and GRE or SPACE) into MRIcroGL - *coregistration should have been done by this point via the SPM toolbox in MATLAB*

****** Note: the MPRAGE used for segmentations should be the nifti file that was converted after running the preprocessing steps in Matlab. If any adjustments (pitch, yaw, roll) need to be made to the MPRAGE via SPM, then that MPRAGE should be used******

- Opening MPRAGE
  - Click *File* → *Open* → *"XXXXXX MPRAGE"*
    - Navigate to the appropriate file path to open image
- Opening GRE or SPACE
  - Click *File* → *Add Overlay* → *"XXXXXX* GRE*"*
    - Navigate to the appropriate file path to open image
    - You can adjust the opacity of the overlay by having the GRE file highlighted and sliding the Opacity bar. Similarly, when adjusting the brightness/darkness of an image, make sure you have the image selected that you want to be altering
- Click *Display* → *Coronal* to give yourself the largest view of the brain

    - You can change the display later to make fine tunings of the segmentations, but having the coronal view open is the most important for the initial drawing
- In MRIcroGL, loading an image automatically sets the parameters for the Darkness and Brightness of the image, which should be good enough for ROI identification. However, if these default settings make it difficult to identify ROIs, then you can manually adjust as necessary

Step 2: Locate the view of the anterior commissure (AC) as seen on slide 22 of the Mai et al. atlas (find the “mustache” of the anterior commissure)

- Scroll anteriorly until you see the anterior commissure
    - The AC should be bulbous in the center, just below the ventricles
        - The fornix should be very prominent, not small and faded away (otherwise you went too far anteriorly)
        - The fornix should not be separated yet (otherwise you went too far posteriorly)
    - Other brain regions should be very noticeable at this view (e.g., the putamen, caudate, internal capsule, globus pallidus) which are great landmarks to set your boundaries
        - Your drawings may be up against these structures, but should not overlap, which will help you find the shape of your drawing

Step 3: Work on segmenting the ROI from slide 22 to slide X (anteriorly/posteriorly), then jump back to slide 22 and work to slide X (posteriorly/anteriorly)

- This allows for an easier segmenting process, as it can be difficult to find ROIs on slides that capture the anterior- or posterior-most portions that might be a little harder to find as a starting point
- Using slide 22 as the landmark allows for an easy reference when moving between multiple slices due to the obvious structure of the AC
- Slide range for ROI’S:
    - BNST: Slides 18-24
    - PVN: Slides 20-28 (20-25 are readily identifiable; 26-28 you risk including other small nuclei)

Step 4: Save the VOI

- Click *Draw* → *Save VOI* → …
    - Save as, “IDnumber_ROI” (i.e., 99999_BNST) to an appropriate file path

Step 5 (angular approach for BNST only): Refine the drawing using GNU Image Manipulation Program (GIMP)

- Open GIMP using the code ‘flatpak run org.gimp.GIMP’ in the terminal
- In MRIcroGL, screenshot your current slice, then paste into GIMP (easiest to work from your first to last slice that includes an ROI drawing)

- Using the measuring tool (Shift + M), measure from the **center of the anterior commissure** (or where it would be, depending on slice) to the corners of your drawing to match that of the following angular measurements (+/- 0.2 degrees, or about 1 voxel):
  - **NOTE:** These measurements are best used as a guide, namely for images where structures are hard to distinguish, or for finding the medial boundaries, as following the shape of known structures (i.e., internal capsule, ventricles, etc.) will provide the most accurate drawing

  - Slice 18:
    - AC to dorsal most BNST: 30.50 degrees
    - AC to ventral most BNST: 9.22 degrees
    - AC to medial most BNST: 16.57
    - Lateral most BNST should be up against the internal capsule, giving the bean shape of the drawing
  - Slice 19:
    - AC to superolateral dBNST: 27.88 degrees
    - AC to inferolateral vBNST: 16.21 degrees (up against EGP)
    - AC to superomedial dBNST: 28.72 degrees
    - AC to inferomedial vBNST: 25.35 degrees
  - Slice 20:
    - AC to superolateral dBNST: 36.23 degrees
    - AC to inferolateral vBNST: 17.29 degrees
    - AC to superomedial dBNST: 38.26 degrees
    - AC to inferomedial vBNST: 23.03 degrees
  - Slice 21:
    - AC to superolateral dBNST: 43.77 degrees
    - AC to inferolateral vBNST: 14.77 degrees
    - AC to superomedial dBNST: 30.07 degrees
    - AC to inferomedial vBNST: 16.28 degrees
  - Slice 22:
    - AC to superolateral dBNST: 44.64 degrees
    - AC to inferolateral vBNST: 21.47 degrees
    - AC to superomedial dBNST: 39.47 degrees
    - AC to inferomedial vBNST: 31.87 degrees
      - Inferomedial arm extent: 38.39 degrees
  - Slice 23:
    - AC to superolateral dBNST: 41.68 degrees
    - AC to inferolateral vBNST: 7.47 degrees
      - Ventral arm extent: 13.31 degrees (runs along ventral pallidum to AC)
    - AC to superomedial dBNST: 34.37 degrees
    - AC to inferomedial vBNST: 16.64 degrees
  - Slice 24:
    - AC to superolateral dBNST: 41.24 degrees (along internal capsule)

        - AC to inferolateral vBNST: 15.20 degrees (along ventral pallidum to AC)
        - AC to superomedial dBNST: 30.14 degrees (against ventricle)
        - AC to inferomedial vBNST: 36.14 degrees (heel next to lower fornix section)

Tips Based on Slice:

- **BNST:**
    - Slice 18:
        - Locate the anterior commissure, which should be two thin lines at this point. Where those two lines would meet at a point is a generally good area for the heft of your BNST drawing
        - Try and locate the external globus pallidus (EGP) and draw a vertical kidney bean shape, which should go slightly beyond the highest and lowest point of the EGP, but will be slightly thinner in comparison
        - This tends to be a hard slice to draw super accurately, as there are no great boundaries that are easily distinguishable. The Mai et al. atlas shows the caudate as being up against this portion of the BNST, but in practice this has not been the case. Angular approach will be more useful for this slice

          s

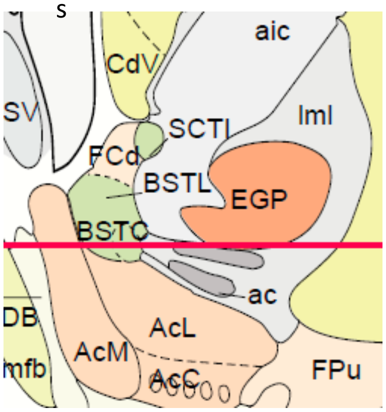

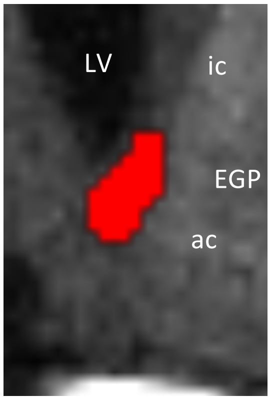

    - Slice 19:
        - Locate the anterior commissure, which should be two lines that meet at a point. The point is roughly the center of your BNST drawing
        - The dorsal extent of your drawing should not go dorsomedial beyond the bottom point of the ventricle and should be up against the internal capsule on the dorsolateral portion. You may also encounter the tsv at this dorsolateral portion
        - The ventral bounds are not as clear, but should run up against the EGP and anterior commissure (angular approach useful here)

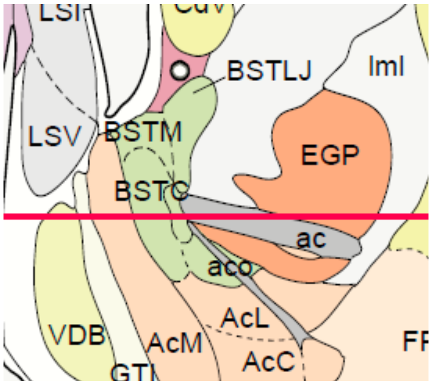

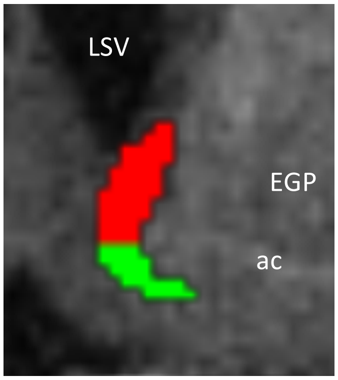

- Slice 20:
  - Locate the anterior commissure, which may be splitting in two, but will still have some heft/a bulbous portion medially
  - Your drawing should extend dorsomedial just beyond the bottom point of the ventricle, but not so far that it is the dead center of the image, and dorsolateral should be up against the internal capsule (potentially the TSV as well)

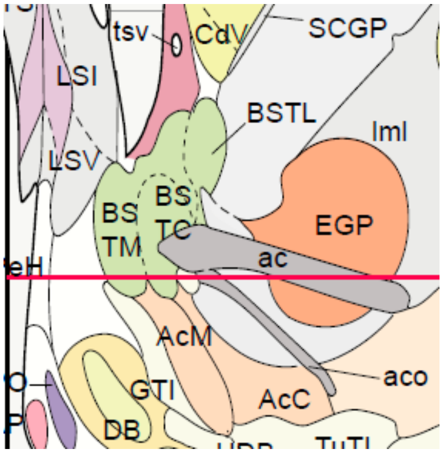

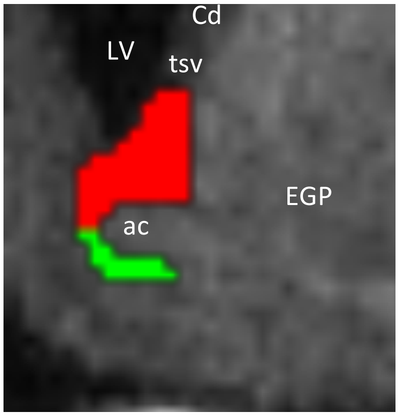

- Slice 21:
  - Locate the anterior commissure, it should be pulling apart from the middle at this point.
  - Your drawing should wrap around the bulbous portion of the anterior commissure and extend dorsolateral to the TSV and internal capsule and dorsomedial beyond the bottom point of the ventricle to the fornix. Ventrally, the drawing should go a few (2-3) voxels below the anterior commissure

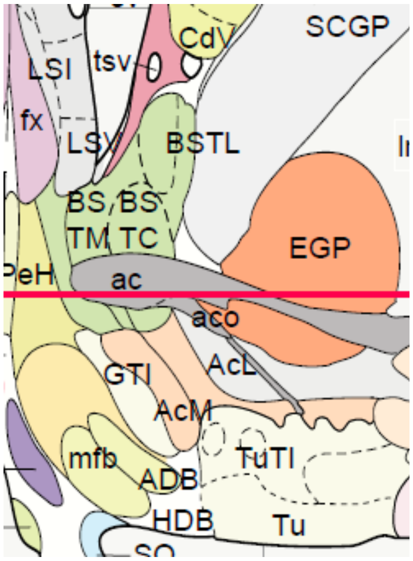

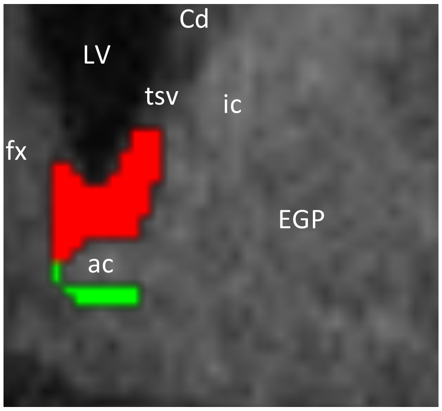

- Slice 22:
  - Locate the anterior commissure, which should centrally have a large bulbous portion visible
  - Your drawing should wrap around the outside and extend dorsolateral to the tsv and internal capsule and dorsomedial to the bottom point of the ventricle. Ventrally, your drawing should extend and wrap around the ventral portion of the globus pallidus out to the lateral portion of the anterior commissure (you can scroll between slices to find the lateral AC if it is hard to see/not visible). Then, draw medially from there to be roughly in line with the outer portion of the anterior commissure bulb (see blue dashed line below)

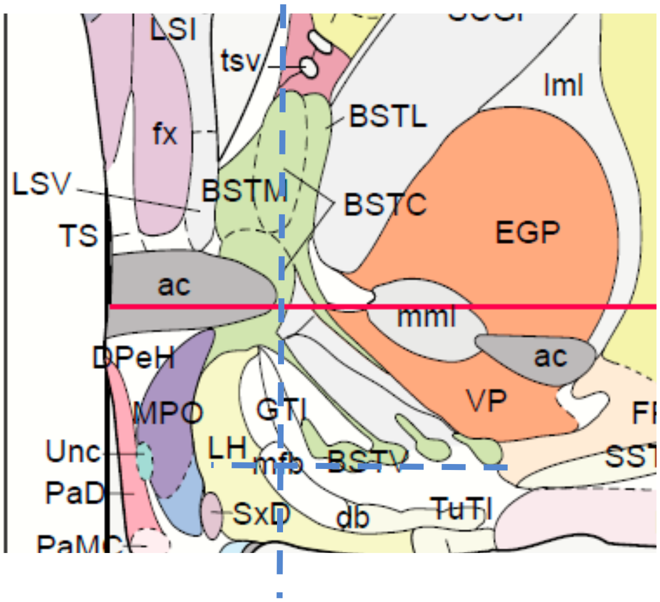

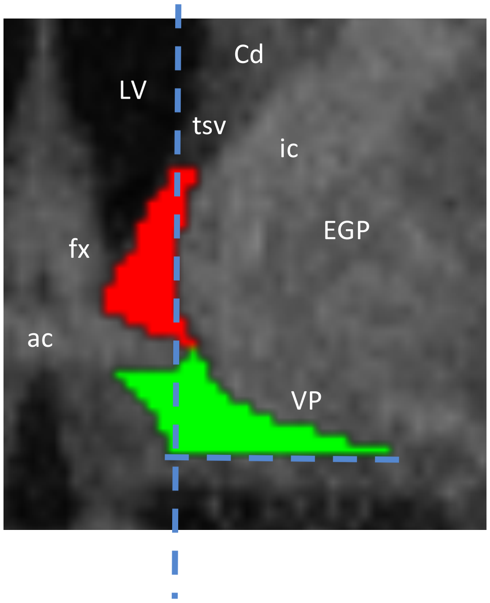

- Slice 23:
  - Locate the fornix (not yet split), ventricle, tsv (if visible), and the internal capsule
  - Your drawing should remain within the bounds of all these structures
  - The ventral arm of the BNST should wrap around the bottom edge of the ventral pallidal area, extending to the anterior commissure, similar to slice 22, but with less inclusion of the medial area under the fornix

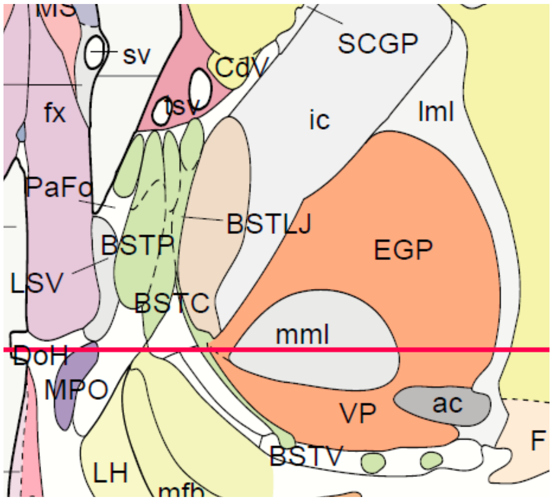

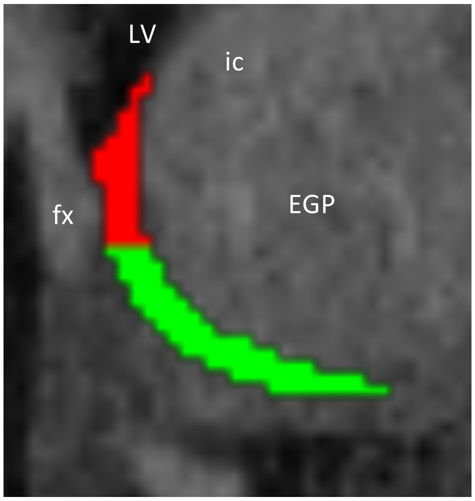

Slice 24:
  - Locate the fornix (should be splitting/split), ventricle, TSV, and the internal capsule
  - Your drawing should be between these boundaries, with a slight heel below the fornix, and a ventral extent that wraps around the pallidal area as seen in the previous slice, also extending to the anterior commissure

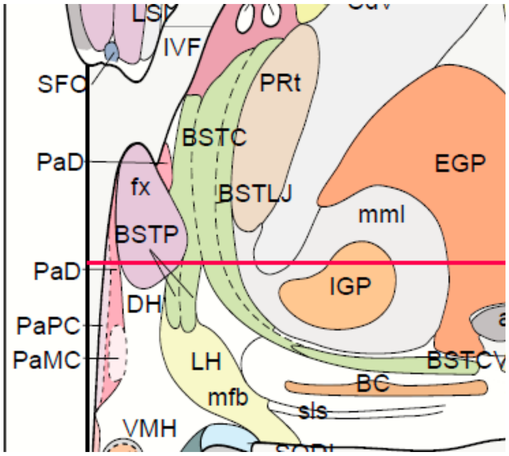

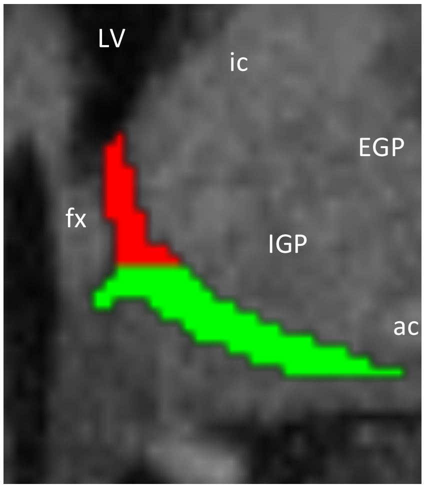

- **PVN (use triangular shapes)**
    - Slice 20:
        - Start the segmentation where the optic tract/optic chiasm (ox) starts connecting to the cortex (see red rectangle on image below)
        - Locate the anterior commissure, which may be splitting in two, but will still have some heft/a bulbous portion medially
        - The PVN (PaAP) will have some overlap with the MPO, but draw a triangle that runs along the 3rd ventricle, medial to the DB, and stays roughly in line with the cortical boundary (blue line)

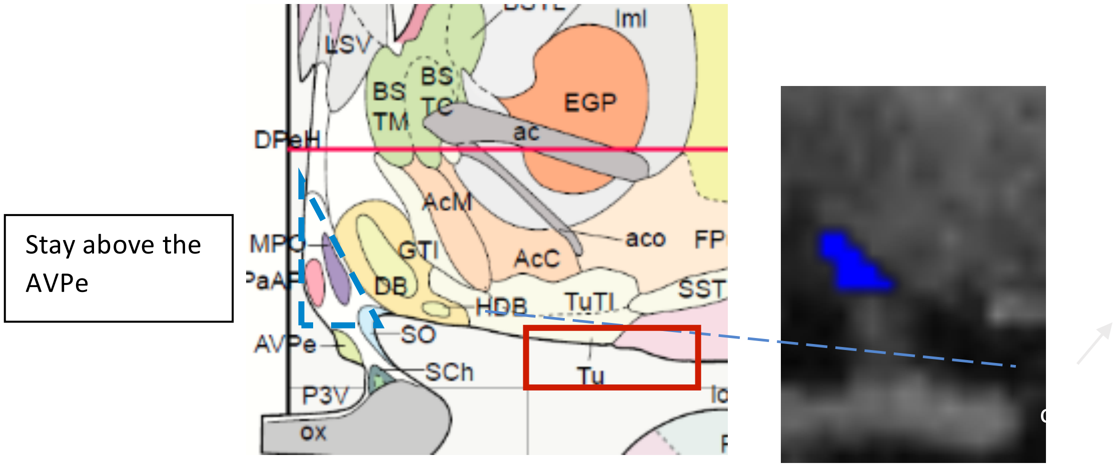

- Slice 21:
    - Follow the same guidelines as before, but you can now move more dorsally toward the anterior commissure and laterally toward the fluid filled space

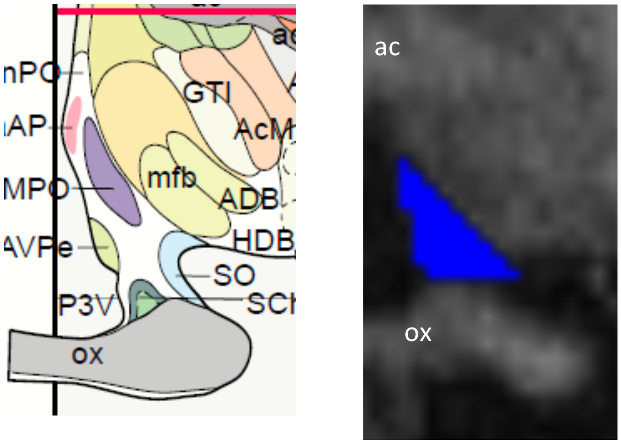

- Slice 22:
  - Follow the same guidelines as before, but you can now move more dorsally toward the anterior commissure
  - Most likely will be up against the anterior commissure at this point

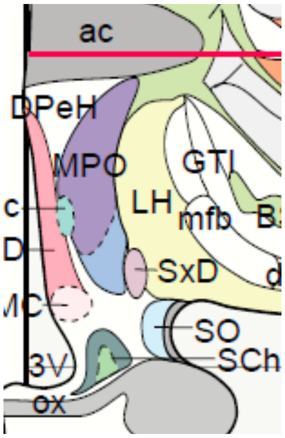

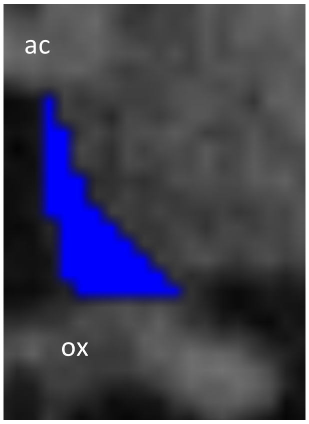

- Slice 23:
  - Anterior commissure should be split from the center now (only more lateral extents are visible), so the lower portion of the segmentation will begin to tuck toward the ventricle, creating a trapezoid-like shape (see below)

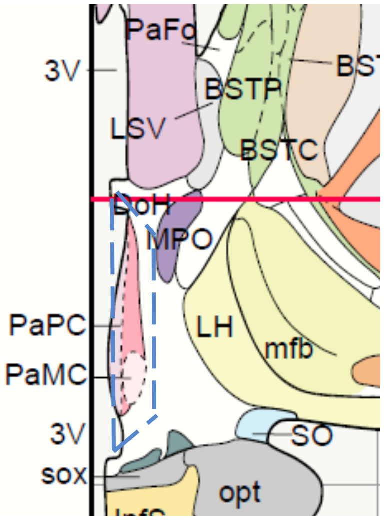

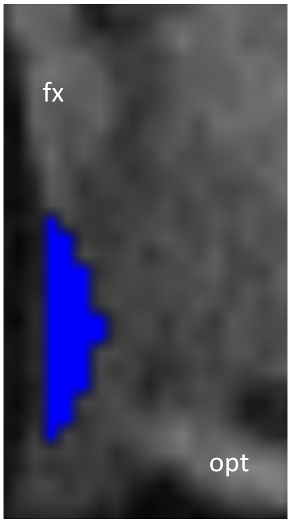

- Slice 24:
  - Now that the fornix is beginning to split, the PVN segmentation will begin "tucking up" toward where the fornix is splitting. Segmentation should remain between the fornix and 3rd ventricle

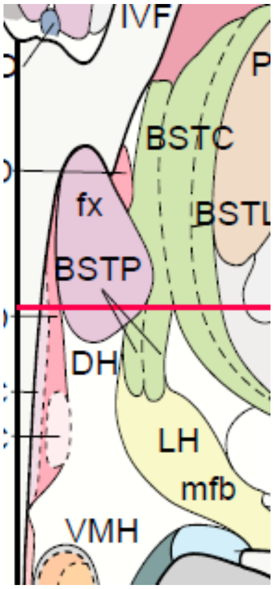

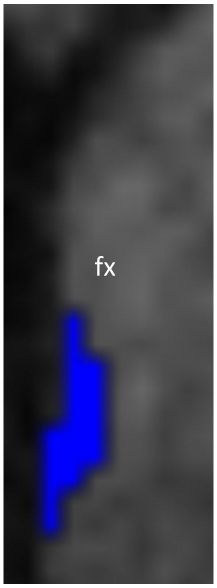

- Slice 25
  - As the fornix splits more (only the little bulb is visible), the PVN segmentation will be smaller as it will be tucked tightly in between the fornix and the third ventricle, slowly moving upward. The bottom boundary of the segmentation should be roughly in line with the bottom of the fornix, or slightly above

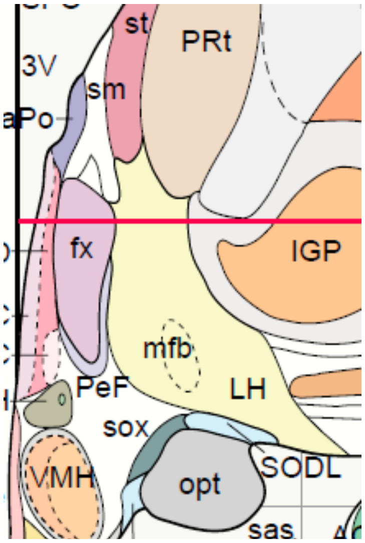

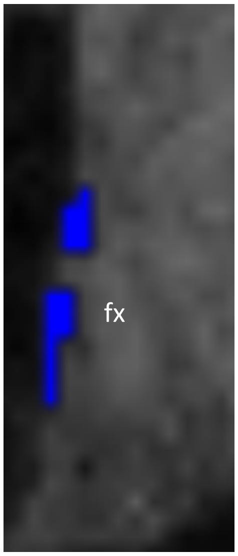

- Slice 26
  - When the fornix is split quite heavily (space above the fornix), the PVN will tuck up and above the fornix. To be safe, it is not worth segmenting this slice, as there are other small nuclei that are difficult to distinguish at this point

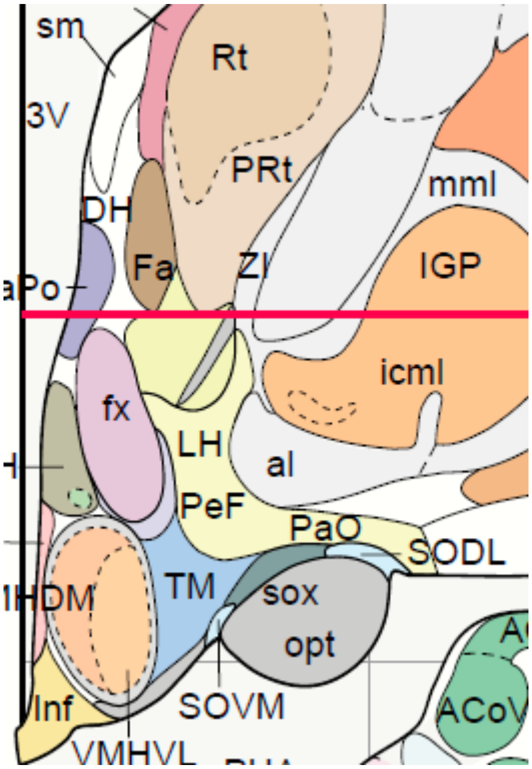

Supplement: Supplementary file 2 — Supplementary file2 (PDF 1769 KB) [file 429_2023_2713_MOESM2_ESM.pdf]
